# EStore: A User-Friendly Encrypted Storage Scheme for Distributed File Systems

**DOI:** 10.3390/s23208526

**Published:** 2023-10-17

**Authors:** Yuxiang Chen, Guishan Dong, Chunxiang Xu, Yao Hao, Yue Zhao

**Affiliations:** 1 School of Computer Science and Engineering, University of Electronic Science and Technology of China, Chengdu 611731, China; 202212081302@std.uestc.edu.cn (Y.C.); 2 Science and Technology on Communication Security Laboratory, Chengdu 610041, China; haoyao30@163.com (Y.H.); yuezhao@foxmail.com (Y.Z.); 3 No. 30 Institute, China Electronics Technology Group Corporation, Chengdu 610041, China; mountain_dong@163.com (G.D.)

**Keywords:** distributed file system, ciphertext retrieval, key management, encrypted storage, fine-grained encryption, secure data sharing

## Abstract

In this paper, we propose a user-friendly encrypted storage scheme named EStore, which is based on the Hadoop distributed file system. Users can make use of cloud-based distributed file systems to collaborate with each other. However, most data are processed and stored in plaintext, which is out of the owner’s control after it has been uploaded and shared. Meanwhile, simple encryption guarantees the confidentiality of uploaded data but reduces availability. Furthermore, it is difficult to deal with complex key management as there is the problem whereby a single key encrypts different files, thus increasing the risk of leakage. In order to solve the issues above, we put forward an encrypted storage model and a threat model, designed with corresponding system architecture to cope with these requirements. Further, we designed and implemented six sets of protocols to meet users’ requirements for security and use. EStore manages users and their keys through registration and authentication, and we developed a searchable encryption module and encryption/decryption module to support ciphertext retrieval and secure data outsourcing, which will only minimally increase the calculation overhead of the client and storage redundancy. Users are invulnerable compared to the original file system. Finally, we conducted a security analysis of the protocols to demonstrate that EStore is feasible and secure.

## 1. Introduction

The Hadoop distributed file system (HDFS) can be used to provide easily available file storage services for massive data within a Hadoop cluster. At present, the HDFS is widely used in various data analysis and storage scenarios such as e-office and the Internet of Things. The Internet Data Center (IDC) predicts that the global dataset created, collected, and copied will reach 175 ZB every year by 2025, an increase of more than five times compared with 2018. A huge data volume requires a big data platform for data management and security protection of sensitive messages such as user identity, user accounts, and biometric features, which, once leaked, will threaten the security of millions of users. It is necessary to provide secure services for the HDFS by means of access control and data encryption.

The existing HDFS has basic resource control abilities, and its control objects are only for HDFS directories and files. Furthermore, most HDFS files are stored in plaintext, whereby its security of access control depends on the configuration of the permission policy. Once the permissions are improperly configured or bypassed, this will lead to disclosure of the data.

Transparent encryption is another commonly used protection method of the HDFS. It protects directories and files by setting up an encryption area. The keys to the encryption area are stored and accessed separately in a key management system (KMS). This is not calculated in real time. Moreover, the keys are not uniformly controlled with file access; therefore, there is a risk that the keys will be leaked. Furthermore, an adversary (including internal and external type) may obtain the keys and decrypt the ciphertext for commercial benefits. Furthermore, if the internal adversary destroys the key, this spoils the availability of the file. The existing KMS only provides a black-and-white list for control, which is very simple and less efficient. Therefore, it is necessary to control the access to both files and keys. The existing key resource and access control methods of the HDFS are coarse; for example, the native POSIX permission model and ACL mechanism only control access to basic operations such as reading, writing, and executing. Apache Sentry provides role-based access control, while Apache Ranger provides label-based (role generalization) access control for the HDFS. None of the existing components provide access control based on fine-grained subject–object attributes. Therefore, it is necessary to design a complete and efficient fine-grained access control mechanism to provide full life-cycle protection of data and keys.

## 2. Related Work

The Hadoop distributed file system (HDFS) has the characteristics of high fault tolerance and high throughput, which are widely used for storing massive data. With the development of Hadoop, its security issues have become increasingly prominent. In order to ensure the security of the data stored in the HDFS, existing methods include access control, encryption, etc.

### 2.1. Access Control

Colombo et al. [1] combed access control models from MapReduce’s distributed computing scenario, including Access Control for the Hadoop ecosystem (HeAC), Object-Tagged Role-Based Access Control (OT-RBAC), and so on. The formal Hadoop HeAC was proposed by Gupta et al. [2], and it describes the authorization mechanism of data in Hadoop services and ecosystem services (such as Hive, Kafka, and other components), and it can set attribute values as tags to control access to various operations. However, they did not consider upload operations or the decision of rejection. Gupta also introduced the mechanism of group hierarchy (GH), proposing that the permissions of Hadoop services can be assigned to certain roles. Awaysheh et al. [3] put forward a federation access control reference model (FACRM) and its implementation process, which is in line with the service-oriented architecture (SOA). Ugobame et al. [4] introduced blockchain into big data access control, complete identity management, and authority control and protected data privacy.

The abovementioned access control mechanisms are set for data in Hadoop without considering the access control for keys. They cannot guarantee the security of data and keys simultaneously and thus cannot prevent the storage server from stealing data. Access control, as the first line of defense for data protection, confirms the identity of the users by adopting authentication methods such as Kerberos, Sentry, and Ranger. However, once the data leave the control domain, protection becomes invalid. On the contrary, data encryption stores and transmits data in the ciphertext, and it provides continuous and stronger protection even if it leaves the access control domain. Below, we review the research on the strength of the data encryption protection effect.

### 2.2. Encrypted Storage

The encryption of Hadoop includes transmission and storage types. In terms of transmission encryption, most components of the Hadoop platform already have mature transmission protocols such as simple authentication and security layer (SASL) and secure sockets layer/transport layer security (SSL/TLS) [5]. In terms of storage encryption, the Apache Hadoop has included a KMS module since version 2.6.0 and a transparent encryption/decryption mechanism, allowing users or administrators to set the encryption area. The Hadoop platform can automatically encrypt data and then transmit and store it when uploading/downloading data to this area. However, the existing KMS has the problems of low search efficiency and low encryption efficiency. At present, the keys are randomly arranged in a hash table according to the hash value of the key name and version number, and the different versions of the same key are also arranged in a disorderly manner, which leads to low key retrieval efficiency. The encryption process built into the Hadoop platform includes plaintext preparation, encryption, and ciphertext sending preparation. These steps are executed in series, leading to insufficient utilization of the encryption resources and low performance. In addition, transparent encryption assumes that the HDFS is maintained by a trusted third party, which is unrealistic; furthermore, the granularity of the encrypted data is too coarse, which also introduces security risks of data leakage. Diaz et al. established an authentication mechanism based on a bilinear elliptic curve in the Hadoop platform, but the computation cost and configuration are relatively complex [6]. Song et al. added the ARIA block encryption algorithm on the basis of the AES algorithm in the HDFS transparent encryption mechanism, making HDFS double encryption algorithms optional [7]. Ciphertext policy attribute-based encryption (CP-ABE) [8,9] and key policy attribute-based encryption (KP-ABE) [10] can indeed provide the functions of encryption and access control simultaneously, but they are essentially public key types, and their overheads are not suitable for the big data environment; furthermore, they require a high degree of freedom for the encryptors.

### 2.3. Searchable Encryption

The HDFS maintained by a storage service provider is supposed to be honest but curious; that is, the cloud server provider honestly performs the storage service for the users; however, it may try to crack and discover the users’ information and privacy. The files are stored in the form of ciphertext, which means that its availability is also reduced when a user wants to locate a specific file in their ciphertext collection stored in the HDFS. If the collections are decrypted and then searched in the HDFS, users’ data may be leaked to the service provider. If the user first downloads their entire ciphertext collection and then decrypts it, it will cause excessive bandwidth usage and computational overhead. This leads to searchable encryption, a method of searching ciphertext with ciphertext, first proposed by Song et al. [11]. Searchable encryption has been widely researched in the context of cloud storage, especially concerning the destruction caused by encryption and the flexibility of searching [12].

In terms of practicality, Golle first established a searchable encryption scheme based on an inverted index using the Bloom filter in 2003 [13]. In 2011, Curtmola first proposed a semantically secure anti-indistinguishable chosen keyword attack (IND-CKA) ciphertext retrieval scheme [14]. Kamara officially put forward the dynamic ciphertext retrieval scheme in 2013 [15]. The dynamic searchable encryption system introduced forward and backward security for the keywords that users have searched. Forward security requires that the cloud cannot learn that newly added documents contain keywords that users have searched. Backward security requires that the cloud cannot learn that the deleted files contain keywords that have been searched for by users.

### 2.4. Comparison and Analysis of Typical Schemes

With the increase in functional diversity and security, the design of the ciphertext retrieval scheme will bring a certain degree of communication and computing overhead. Table 1 shows a comparison of a typical technical route of encrypted storage and secure data sharing. We can see that a fair amount of encrypted storage has been realized based on attribute-based encryption (ABE) [16,17], broadcast encryption (BE) [18], and homomorphic encryption (HE) [19]. However, they cannot adapt to large file encryption due to the computational storage overhead. The remaining schemes [20,21] lack data security sharing methods and are not suitable for the actual file system scenario [22].

In terms of practicality, it is necessary to comprehensively consider safety, function, and efficiency. With the increase in application requirements, the development of ciphertext retrieval has also shown diversity, for example fuzzy keyword search, searchable encryption based on order-preserving or order-revealing encryption to support the range query, the use of homomorphic algorithms to realize Boolean search to improve security, realizing multi-keyword ciphertext retrieval based on vector space, retrieving results with relevance ranking by considering the correlation of ciphertext keywords, and so on [23,24,25,26,27,28]. The above schemes all assume that the cloud server is “honest but curious”. Further, under the assumption that the cloud server is malicious, the cloud service provider may tamper with the search results driven by interests. In response to this risk, verifiable-based encryption schemes could verify the correctness and integrity of the cloud storage files [8,29,30,31].

In addition to ciphertext retrieval, private information retrieval (PIR) and oblivious random access machines (ORAMs) support privacy-preserving searching [32,33], but they are fundamentally different from searchable encryption. Private information retrieval includes the sender and the receiver. The sender holds the data, and the receiver holds the index; thus, the sender can let the receiver know the specific data without obtaining any additional information. However, the stored data are in plaintext, and therefore confidentiality cannot be guaranteed. ORAMs can be used to support all functions of ciphertext retrieval, where the server only knows the size of the file set, thus providing a higher security level. Furthermore, reducing the communication and storage overhead is a difficult problem.

Public key searchable encryption (PSE) adopts asymmetric cryptography [34], which is suitable for the scenario where the sender shares their outsourced data with the receiver in the cloud. Boneh put forward the first public key searchable encryption scheme in 2004. Because public key searchable encryption is inherently suitable for shared scenarios, it is easier to expand to many functions compared to the symmetric type, including multi-keyword queries, equality queries, range queries, and subset queries [23,24,25,26,27,28]. Meanwhile, most public key searchable encryption systems are designed based on bilinear pairing, which is less efficient than the symmetric type; furthermore, its design mostly focuses on the text file scenario, lacking the design of complex data structures.

In the construction of public key searchable encryption, the sender generates a different encrypted keyword index for the receiver based on random numbers, which can easily maintain forward and backward security. However, the adversary can easily obtain the receiver’s public key to become the sender, then they can carry out subsequent keyword guessing attacks (KGAs) [35]. Countermeasures include authorizing the cloud, preventing the cloud from carrying out keyword guessing attacks based on indistinguishable confusion and signcryption. Furthermore, fuzzy keyword search, an independent entity server that assists users in generating the index, can also prevent KGAs. Regarding public key management, public key infrastructure (PKI) or key generation center (KGC)-based certificateless cryptographic systems can be adopted [18]. With the development of quantum computers, public key searchable encryption schemes based on quantum cryptography are also a research hotspot.

The combination of PSE and other cryptographic techniques mainly includes identity-based encryption (IBE), functional encryption, differential privacy, and homomorphic encryption [19,36,37]. The first PSE proposed by Boneth is based on IBE [38]. With the identity as the keyword, its future direction may be searchable encryption with anonymous identity. Function encryption supports restricted key use, and therefore the receiver can only obtain the file results processed by a specific function without revealing other information. In addition, the characteristics of homomorphic encryption can support SE, but both homomorphic and functional encryption are limited by the excessive computational and storage overhead [19].

### 2.5. Requirement Analysis and Our Contributions

According to the above situation and security risks, big data security protection needs to meet the following requirements:

(1) Key resource control: the keys stored and managed in a centralized way need effective access control (authentication).
(2) Normalized description of operational semantics: Access control policies need normalized description, authentication, and unified management functions.
(3) Fine-grained access control: mechanisms should have fine-grained access control for files and keys to protect who and when in what scenario, from what equipment, via what network, through what operation, and accessing what data.

Specifically, our contributions are as follows:

- We propose a user-friendly encrypted storage scheme named EStore based on our previous work [39], which mainly consists of file encryption and ciphertext retrieval. In EStore, users protect keywords and file data by encryption when outsourcing. The scheme ensures that only authorized users can reconstruct readable data from ciphertext; furthermore, the scheme supports hierarchical encryption to enhance security.
- We combine ciphertext computing, including searchable encryption and file encryption, with a distributed file system, designing reasonable key distribution and derivation mechanisms to support the application of secure sharing and exchange of ciphertext data.
- Our performance evaluation and security analysis shows that EStore is secure, inexpensive and efficient. The application of encryption and index construction before files are uploaded guarantees confidentiality. The key derivation mechanism removes the need to manage a large number of keys, which is more efficient and safer.

The rest of the paper is organized as follows: In Section 2, we present the system model and threat model. In Section 3, the protocol designs are demonstrated, and the designs of the main cryptographic algorithms are outlined in Section 4. Then, we evaluate the system performance and analyze system security in Section 5. Finally, we draw our conclusions in Section 6 and discuss the future directions of the research in Section 7.

## 3. The System Model and Threat Model

### 3.1. System Model

**Figure 1:**
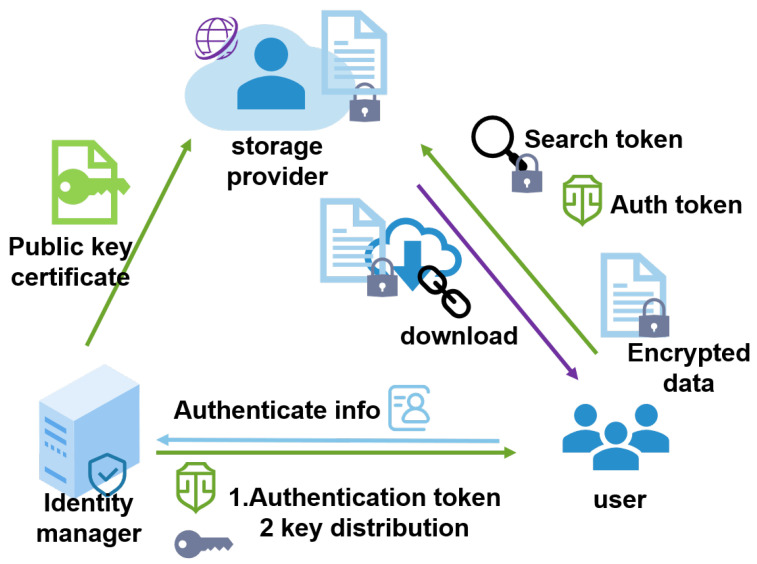
Encrypted storage system model.

Considering the requirements analyzed above, the cloud service providers are supposed to be “honest but curious”. Based on the principle of responsibility separation, we separated the key management and handed it over to a trusted center for management. This may be a company who rent for cloud storage but manage the key center on their own to minimize data leakage. The encrypted storage system model is shown in Figure 1. It depicts three entities: the storage provider, identity manager, and users.

1. The storage provider queries the identity manager for their public key certificate, verifying the authorization token issued by the identity manager. Furthermore, it provides storage and search services for the users.
2. The identity manager is a trusted third party, they authorize the users’ identities when registering and authenticate users when they log in. Furthermore, the identity manager also distributes relevant keys in the user registration, authentication, and file sharing, providing their public key for the storage provider to verify their issued token.
3. Users initiate registration and authentication with the identity manager, receiving the corresponding keys from the identity manager. When uploading and downloading, the users use their file keys to encrypt the plaintext and decrypt the ciphertext, use their searchable encryption keys to generate a cipher index when uploading, and generate encrypted search tokens when searching.

Here, we briefly describe how the model works. In a typical file system, different users collaborate with each other with the help of a storage provider; that is, when user A sends files to user B, the cloud will automatically back up the files for participants to download. This means that the shared files are out of the user’s control, and the data can be stolen by the storage provider; therefore, the users need to encrypt the files before uploading them. Meanwhile, users need to generate a ciphertext index and upload it with the encrypted files for the subsequent secure search. The related encryption and search keys are obtained after the user registers and authenticates with the identity manager. If the user authenticates successfully, they will receive a token with the signature of the identity manager; this can be verified by the storage provider using the issuer’s public key.

### 3.2. Threat Model

To better demonstrate the threat analysis, we divided the threats into two categories as follows: internal adversary and external adversary. They may obtain access rights from the users and storage provider, respectively.

An internal adversary is a legitimate participant in our system model who performs their own behavior according to the preset rules. However, they may be driven by alternate interests, trying to obtain the rights of other participants based on the existing rights, including data, behavior privacy, etc.

For example, the storage provider is supposed to be semi-trusted and provide users with storage services as agreed; however, they might attempt to steal the users’ data and analyze their search behavior privacy. Furthermore, a legitimate user may try to steal other users’ data and privacy based on their own authority, which is beyond their privilege.

An external adversary mainly refers to man-in-the-middle attacks in communication between entities. Such an adversary may intercept and decipher the transmitted information; furthermore, they may impersonate legitimate entities to communicate, steal data, and send fake messages.

To ensure the security and efficient in a distributed file system, a user-friendly encrypted file system scheme should achieve the following goals:

Function:The identity manager can provide registration and authentication for users. Users can protect their uploaded data and directly query their ciphertext.

Security: The identity manager can control the identity and access behavior of users. Users can collaborate safely, not worrying about storage leakages or transmission leakages.

Efficiency: the protection measures will not significantly increase the overhead, including the computation and storage overhead of the client and the cloud storage provider.

### 3.3. System Architecture

Encrypted storage has been widely studied, such as semantically extensible searchable encryption for encrypted storage [40], retrieval schemes in edge computing [29], attribute-based encryption for the HDFS [10], and key management structure [41]. However, previous work has only focused on specific points, and we are not aware of a systematic design for actual big data platforms.

EStore improves the ability of existing file systems [10,29]; further, we took into account both the confidentiality and usage of data, and we performed a system evaluation to provide a higher practical reference value than other schemes [17,19,21].

Our scheme mainly provides data encryption protection, ciphertext search, and secure data sharing for users, and encryption protection guarantees that users can totally control and change the encryption keys, so as to generate encrypted indexes together with files then upload them to the cloud server, like a “key–value” pair. The ciphertext search guarantees that users can generate encrypted search tokens for the server to locate the position of encrypted file. Secure data sharing means one user can directly share their file with another as long as their attribution is in accordance with the access control strategy.

**Figure 2:**
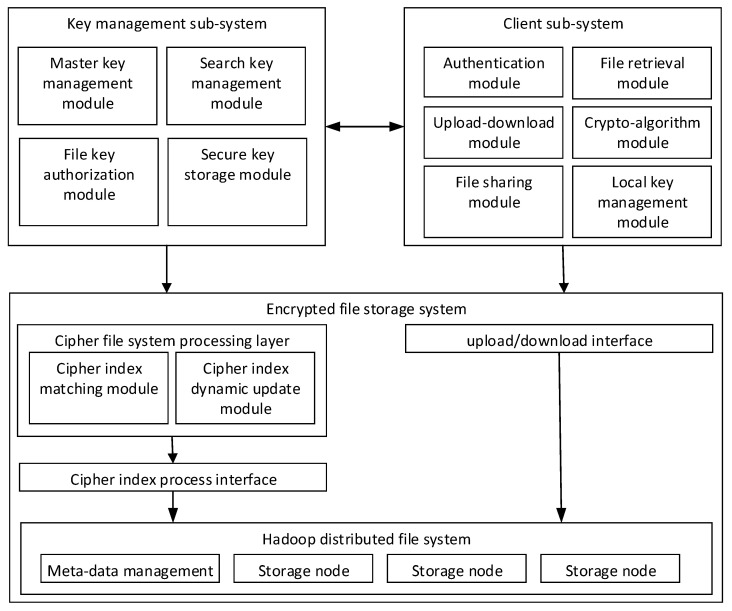
System architecture.

The structure of the encrypted storage system is shown in Figure 2. It mainly includes the key management subsystem, client subsystem, and encrypted file storage system.

- The key management subsystem (regard as a trusted third party) provides search keys, authentication keys and matter keys for the clients when users register and authenticate. In the process of file sharing, key management securely distributes file keys to the shared users and endorses their cooperative behaviors.
- The client performs encryption before uploading and decryption after downloading the files. The client uploads the encrypted files together with a cipher index list, which is used for location in the storage provider. When the user executes sharing operations, they deliver the encryption key to the client of the shared user through a secure channel and notifies to receiver through the key management subsystem (endorsement). The shared user needs to confirm the notification and update their ciphertext keyword index list on the storage system.
- The encrypted file storage system (storage provider) obtains the public key and retrieves the key from the trusted third party, maintaining an encrypted index list for the users. It also provides the user with storage, updates and queries of their ciphertext.

## 4. Protocol Design

### 4.1. Design Goal

**Figure 3:**
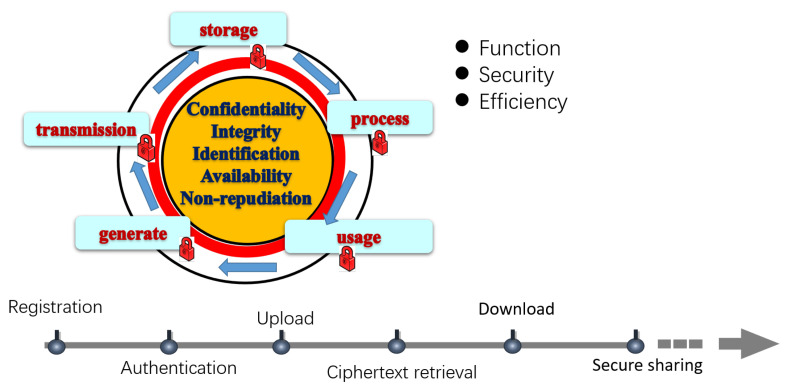
Macro security perspective of the protocol design.

In addition to the above functions, security, and efficiency mentioned in the threat model, our design goal focuses on the whole life cycle of data, shown in Figure 3.

We divided the whole life cycle of data into generation, transmission, storage, processing, and usage; each phase has the requirements of confidentiality, integrity, identification, availability, and non-repudiation. Further, we designed six protocols to cover the users’ safe and friendly use of the file system as follows:

Registration: The identity manager registers the users’ identity and assigns the relevant keys to the users based on the security parameters.

Authentication: Authentication guarantees the users’ access to their own identities and keys.

Upload: The client ensures that the uploaded file has been fully encrypted and the corresponding ciphertext index is established.

Ciphertext retrieval: Ciphertext retrieval ensures that the user-input keywords are not leaked.

Download: Downloading ensures the received ciphertext can be correctly decrypted.

Secure sharing: Secure sharing ensures accurate authorization of the file keys and encrypted files.

Table 2 shows the notations and descriptions used in our protocols.

### 4.2. User Registration Protocol

**Figure 4:**
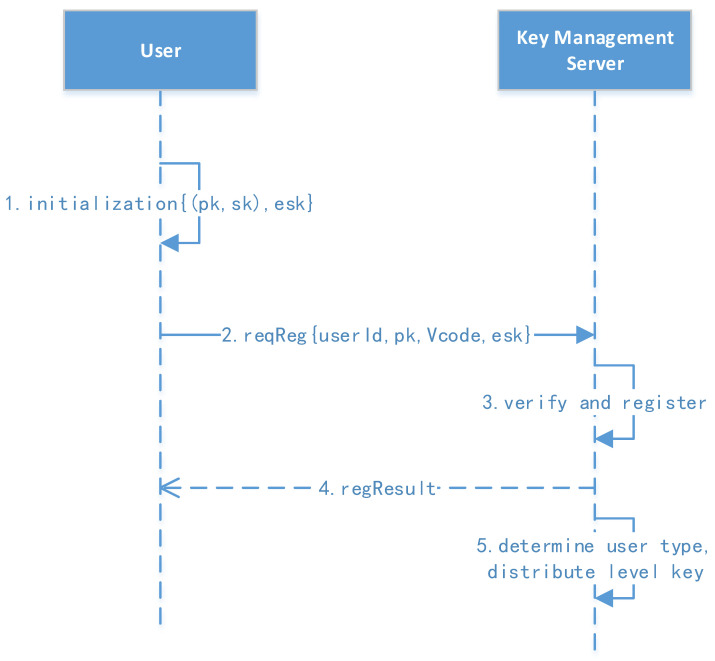
User registration protocol.

Figure 4 shows the user registration protocol.

1. User initialization and registration: The user’s background client first generates a public key (pk) and a secret key (sk). The user manually enters the userID, verification code, and password, of which the verification code can be initialized in batches by the administrator in advance.
2. Initiate the request: The user computes the message (msg=$E_{pwd}\left(sk\right)∥pk∥VerifyCode∥$
  Time∥Sign, $Sign=E_{sk}(Hash(E_{pwd}\left(sk\right)∥pk∥VerifyCode∥Time))$) then sends the message to the key management server for registration.
3. Verify and register: The key management center verifies the signature and registration. The parameters $E_{pwd}\left(sk\right)$, pk, $E_{pk}\left(LK\right)$, and $E_{pk}\left(RK\right)$ are saved, where RK (32 bytes) is the user’s root key generated by the key management server. The user status is then changed to an active status.
4. The registration result is returned to the user.

### 4.3. User Authentication Protocol

**Figure 5:**
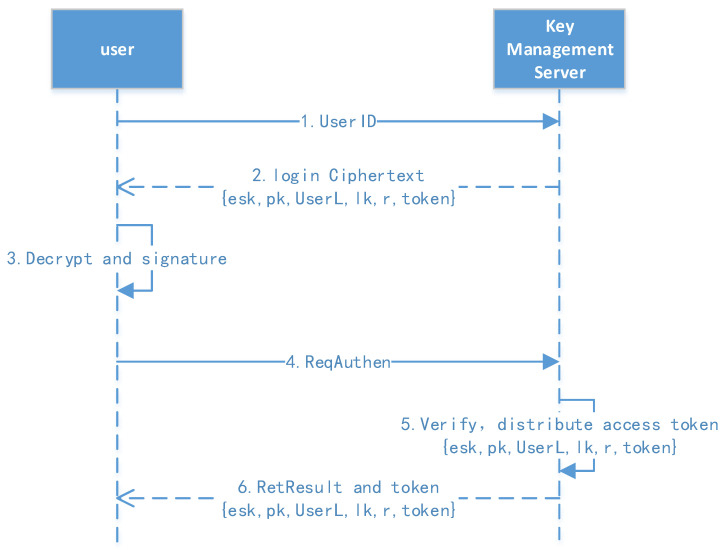
User authentication protocol.

Figure 5 shows the user authentication protocol.

1. Request for stored information: The user first sends their ID to obtain the stored information, including esk ($esk=E_{pwd}\left(msk∥sk\right)$), pk, tag, lk (level key), r (random number), token, etc.
2. Decrypt and request authentication: The user decrypts the stored information, obtaining the msk and sk, then they compute msg=ID∥Time∥Sign and initiate the authentication request.
3. Verify and return the result: The authentication server verifies the user’s signature and distributes the access token; the token’s validity can then be configured.

### 4.4. File Upload Protocol

**Figure 6:**
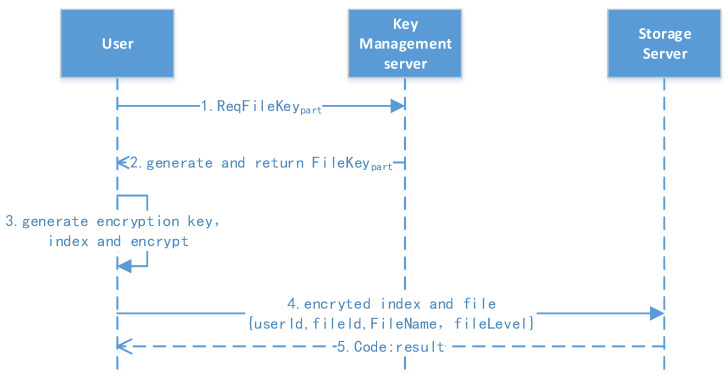
File upload protocol.

The file upload protocol is shown in Figure 6. At this stage, the user has already logged in, and they have a user profile, which includes rk, pk, sk, lk, etc.

1. Request for partial key: The user extracts the file ID and requests the partial key to the file.
2. Encrypt and upload: The user generates the file key (filekey) using lk, r, and the file ID combined with the sm3 hash function. They then encrypt the file and generate the file index, combining the message and upload after the signature. The server returns an “Ok” message if the update is successful.

### 4.5. Ciphertext Search Protocol

**Figure 7:**
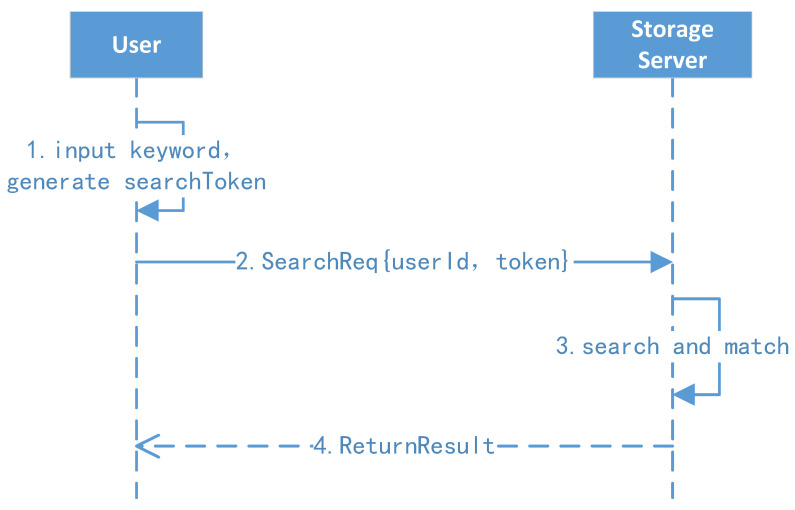
Ciphertext search protocol.

The ciphertext search protocol is shown in Figure 7.

1. Client search and request: The user enters a keyword w, and then the client background generates the search token $t=\left(E\left(w\right),k\right)=\left(\pi_{K_{3}}\left(w\right),f_{K_{2}}\left(w\right)\right)$ and initiates a search request to the search server.
2. Server search: The search server uses the search token t to find the first node’s position and key $k_{0}$; after this, the server can extract all identifiers of the keyword one by one and return the results to the user.

### 4.6. File Download Protocol

**Figure 8:**
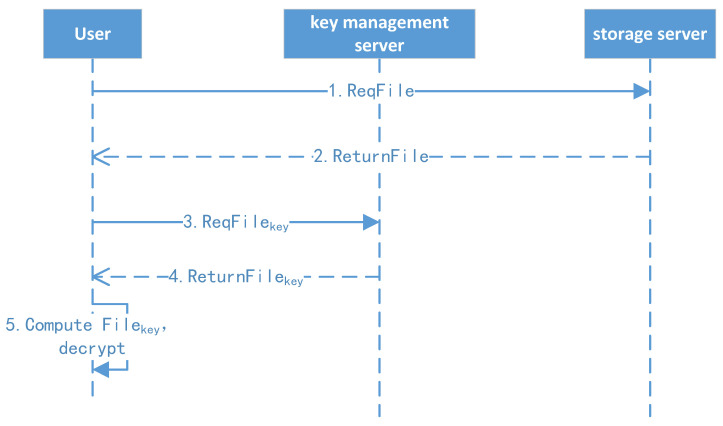
File download protocol.

The file download protocol is shown in Figure 8.

1. File download: The client initiates the download request to the file storage system and downloads the file.
2. File decryption: If the file is first shared from other users, the client uses their root key, CA (shared key), file level, and file ID to derive the file key (FK). Otherwise, the client uses their master key, level key, and file ID to compute the encryption key (FK). Finally, the client decrypts the file, and the details are shown in the following key management algorithm and file classification encryption/decryption algorithm.

### 4.7. File Secure Sharing Protocol

**Figure 9:**
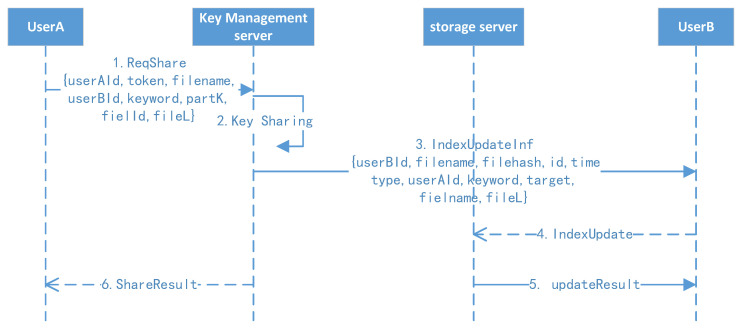
File secure sharing protocol.

The file securing sharing protocol is shown in Figure 9.

1. Active sharing: User A randomly selects another user (suppose B) they want to share with, clicking the file with keywords. User A’s client background will compute the file key and initiate the share operation. A notification will be sent to user B.
2. Receive sharing: After receiving the notification, user B will click the confirm button, and their client background will refresh their own index to finish the receiving operation.

## 5. Main Cryptographic Algorithm

### 5.1. Key Management Algorithm

**Figure 10:**
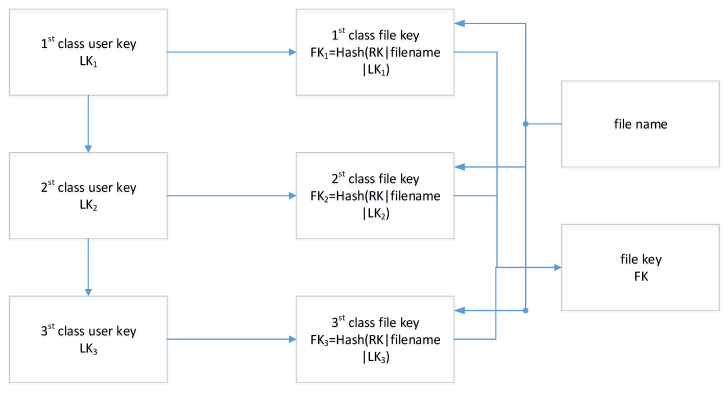
The logic of key management.

The logic of key management is shown in Figure 10. Suppose SKE is a symmetrical encryption algorithm including initialization, encryption, and decryption; Hash is a one-way trapdoor function, and the steps of key derivation related to file encryption/decryption are as follows:

1. Initiation: The key management center (KMC) generates the public parameter param and master key MK.
2. Registration: User $U_{i}$ initiates registration with their identity and attributions, and KMC computes the root key RK for the users to issue a certificate including the corresponding public key PK for the user. Furthermore, the KMC issues a grade key $LK_{u}$ (u = 1, 2, 3) to the user according to their grade level. The level keys are divided into different hierarchies from top to bottom, a first-level key generates a second-level key through a pseudo-random function Rand by computing $LK_{2}=Hash\left(LK_{1}|2\right)$, and a third-level key can be obtained in the same way by computing $LK_{3}=Hash\left(LK_{2}|3\right)$, of which | means concatenation.
3. File key derivation: Files belonging to users have different security divisions, which means the files need to be differentiated when uploaded. A file key FK can be computed by $FK=Hash(RK|filename|LK_{u})$, u = 1, 2, 3, corresponding to its authorized level. Furthermore, the secret key SK for ciphertext retrieval can be computed by SK=Hash(RK|nonce), of which nonce is a random string. Finally, the background of the client computes $C_{K}=Encrypt\left(FK,hash\left(password\right)\right)$ and stores $C_{K}$ as a credential in the key management system, wherein the password is set manually.

### 5.2. File Hierarchical Encryption/Decryption Algorithm

Our scheme uses a digital envelope to secure the key transmission, and the confidentiality of the file is guaranteed by symmetric encryption, while the key of symmetric encryption is guaranteed by public key encryption. Furthermore, the index of the encrypted file is processed by searchable encryption. We take the national algorithm of China, SM2, as an example to demonstrate the efficiency.

For example, a user has a file with security level X. They first calculate the file key as is shown in the key management algorithm ($FK_{X_{u}}=Hash(Rk|filename|LK_{u})$). Then, they encrypt file A with file key $FK_{X_{u}}$ by computing $C_{F}=Enc\left(FileX,FK=FK_{X_{u}}\right)$.

Finally, the client background encrypts the file key with the user’s password key K=hash(password) by computing $C_{K}=Enc\left(FK_{X_{u}}\right)$ and stores $C=(C_{F},C_{K})$ in the HDFS.

In the decryption phase, the user decrypts the file key by computing $FK_{X_{u}}=Dec\left(C_{K},K\right)$. Then, they can restore file key $FK_{X_{u}}$ corresponding to their authorization and compute $Dec(C_{F},FK_{X_{u}})$ to obtain the file.

### 5.3. Searchable Encryption Algorithm

**Figure 11:**
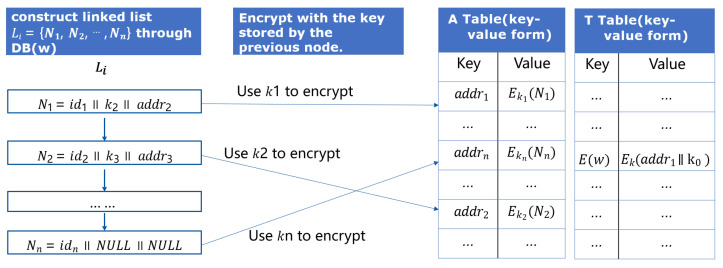
Structure of the search index.

The core of the ciphertext search protocol is to locate the unique identifications of the files through keywords, so as to provide users with download links. The key to accurate positioning lies in how to construct the ciphertext index. Figure 11 shows the structure of our search index I=(A,T) maintained by the server, which includes Table A and Table T for a keyword w; the server establishes the mapping of the keyword to several files (including this keyword), which is called Table A. Suppose the files’ unique identifiers are $DB\left(w\right)=\left(id_{1},id_{2},...,id_{n}\right)$. Then, the linked list $L_{i}=N_{1},N_{2},...,N_{n}$ is constructed based on these files DB(w), each node $N_{i}=id_{i}∥k_{i+1}∥$ including the current file identifier $id_{i}$, key $k_{i+1}$ to encrypt the next node $N_{i+1}$, and the node $N_{i}$ itself encrypted by key $k_{i}$, which is stored in previous node $N_{i-1}$. The position of the first node and its encryption key $k_{0}$ are encrypted and stored in the constructed Table T, which is also in the form of a key–value pair.

## 6. Deployment and System Test

### 6.1. Deployment

**Figure 12:**
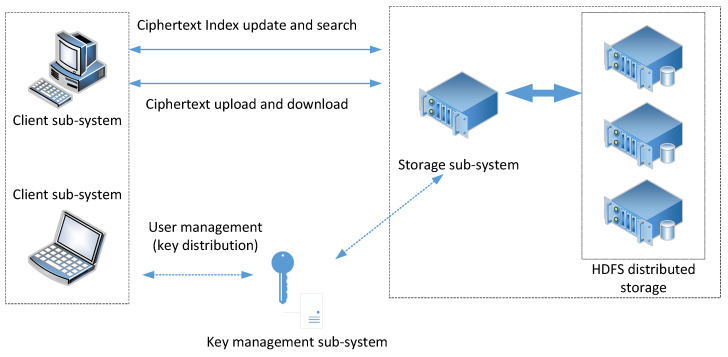
Deployment of the cipher search system.

The test environment includes two Windows terminals and five Linux servers (shown in Figure 12), and these devices are connected through the network. The Windows terminals are used to deploy the client subsystem, two Linux servers are used to deploy the key management subsystem and storage service subsystem, and the remaining three Linux servers are used to deploy the HDFS to provide a distributed storage resource pool.

The environment simulates the typical-use environment of the file system, where users can access the storage service through the client subsystem (including client software and software development kit), and the key management subsystem supports user management and related key management. Its corresponding configuration is shown in Table 3.

### 6.2. System Loss Test

In order to compare the loss of the system caused by the increase in the system overhead after the addition of the protection measures, we tested the average upload speed of files uploaded through the SDK of the client (V) and the average upload speed of files uploaded directly through the HDFS API (V0).

We prepared a group of samples of typical files of different sizes, that is, randomly generated files of 1 MB, 5 MB, 10 MB, 20 MB, 40 MB, 50 MB, 70 MB, and 100 MB; the total size of each group of file samples is no less than 1 GB. We tested the data upload efficiency with protection measures (including ciphertext computing) and called the client SDK to upload the sample groups of different sizes. Each test uploads all files in the same group, and we recorded the total file size and time of the upload; furthermore, we calculated the upload velocity of the sample group.

For comparison, we directly called the API of the HDFS to upload the above test samples of different sizes (without protection measures). Similarly, we recorded the total file size and time and then calculated the average upload velocity of the sample groups. Finally, we compared the average upload velocity of calling the client SDK upload and HDFS API direct uploading.

**Figure 13:**
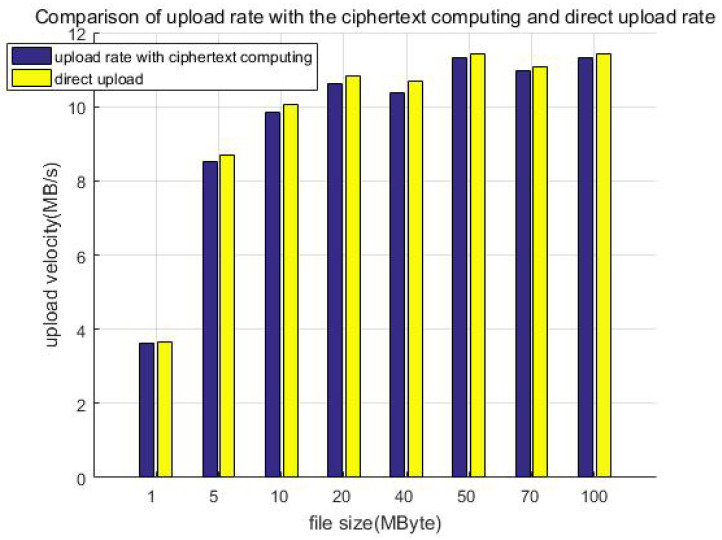
Comparison of upload rate with the ciphertext computing and direct uploading.

Figure 13 shows the comparison of the upload rate with the ciphertext computing and direct uploading. When the file size is less than 50 MB, the upload velocity gradually increases as the file size increases. When the file size exceeds 50 MB, the upload velocity is stable at about 11 MB/s as the file size increases, which is related to the network bandwidth of our equipment. This can be improved by adopting advanced equipment, for example, gigabit optical fibers. Furthermore, we can see that the direct upload is slightly faster than uploading with protection measures (ciphertext computing). This is because the client encryption procedure generates additional calculation overhead, which slows down the upload velocity. We call this part the system loss after adding the protection measures and define the loss as in Equation (1), calculating the system efficiency:

(1) $$\begin{array}{c} \frac{V0-V}{V0}=1-\frac{V}{V0} \end{array}$$

Table 4 shows the upload velocity of the method with protection measures, direct uploading, and upload speed ratio. We constructed file data with different sizes but similar total data amount (1 GB) as test samples to test the upload velocity, for example building 1000 files of 1 MB in size, 200 files of 5 MB in size, or 100 files of 10 MB in size. Although the file size is different, it is multiplied by the corresponding number to make the total data amount of each test sample the same (about 1 GB in each column). It can be seen that the ratio of the upload speed is greater than 70%; that is, the system efficiency loss is less than 30%.

**Figure 14:**
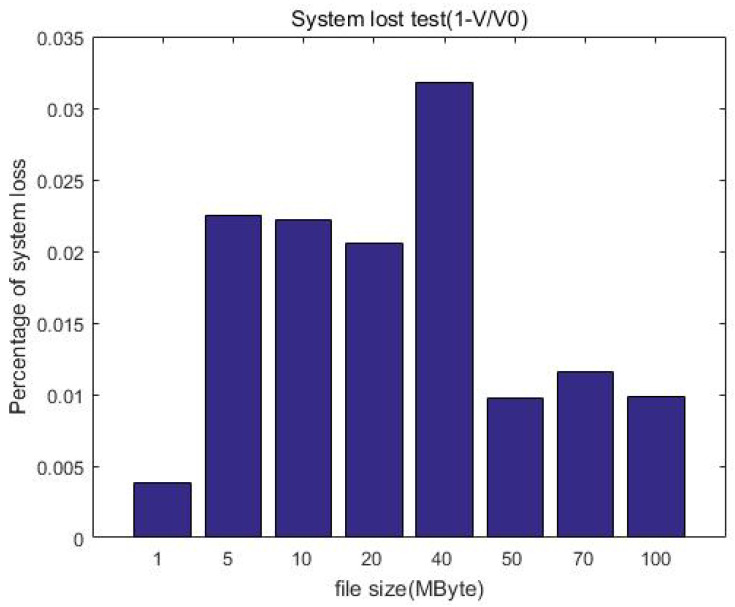
System loss after the protection measures.

Figure 14 shows the changes in system loss with the increase in file size. Compared with the unprotected case, the increase in computational overhead for increasing the protection measures is constant (o(1)); this means the system loss is acceptable and will not affect the user experience when uploading.

### 6.3. Storage Redundancy Test

In order to compare the redundancy increase in ciphertext storage with ordinary storage, we uploaded files through the client software development kit (SDK) to obtain the total size of the ciphertext (S1) and compared the total size of corresponding uploaded plaintext in the pure HDFS (S0, ignoring the size of the HDFS metadata). Therefore, we can define the redundancy as Equation (2), this denotes the size increase ratio of the uploaded data after the protection measures.

(2) $$\begin{array}{c} \frac{S1-S0}{S0}=\frac{S1}{S0}-1 \end{array}$$

**Figure 15:**
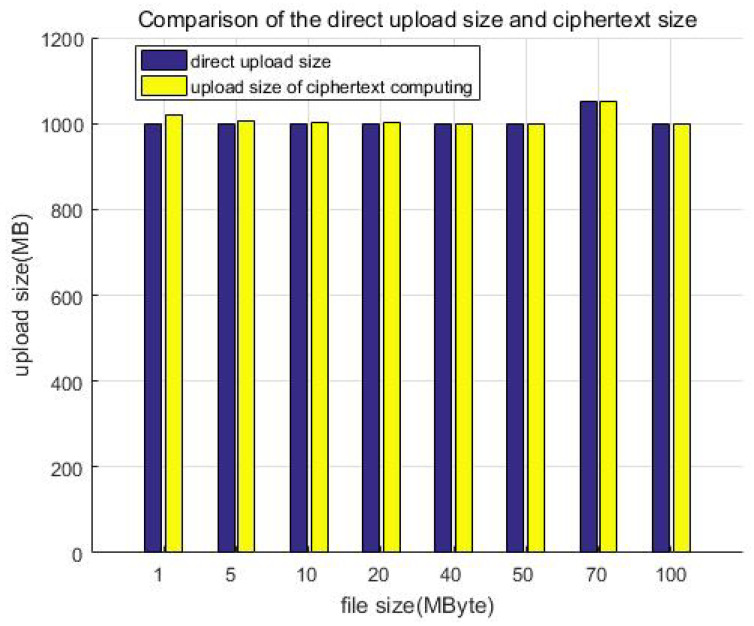
Comparison of the direct upload size and ciphertext size.

Table 5 shows the storage size of the ciphertext, plaintext, and their comparison, while Figure 15 shows the comparison of the direct upload size and ciphertext size. Similarly, we constructed file data of different size but a similar total amount (about 1 GB) as test samples to test and compare the storage size. For example, building 1000 files of 1 MB in size, 200 files of 5 MB in size, or 100 files of 10 MB in size. Although the file size is different, it is multiplied by the corresponding number to make the total data amount of each test sample the same (about 1 GB in each column). It can be seen that the size increase ratio of the uploaded data is less than 10% after encryption; that is, the storage redundancy efficiency loss is less than 10%.

**Figure 16:**
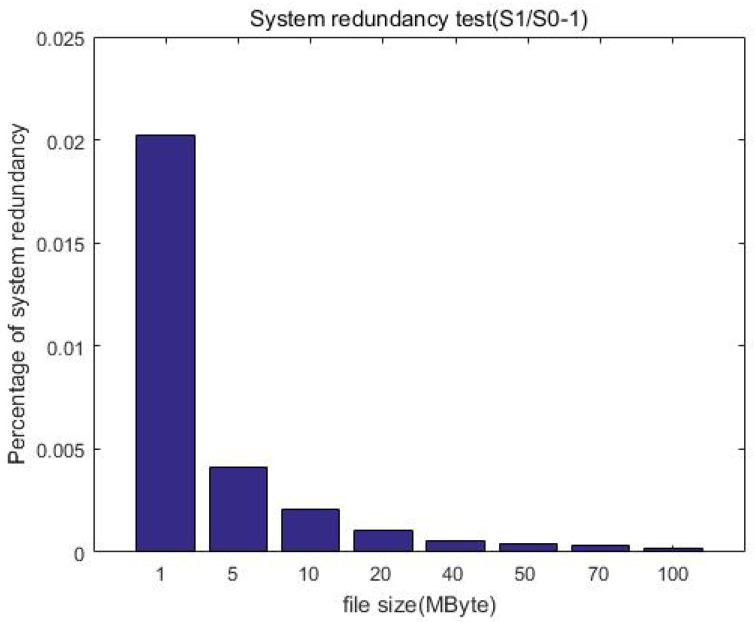
System redundancy test after the protection measures.

Figure 16 further shows that the redundancy gradually decreases with the increase in file size. This is expected because the file size before encryption is equivalent to that after encryption, and thus the redundancy is mainly the increase generated by the ciphertext index, that is, the metadata of the ciphertext. When the uploaded file is small, it may be equivalent to corresponding ciphertext metadata, and thus the redundancy is high. When the file size gradually increases, the generated ciphertext index size is unchanged, and thus the redundancy is reduced. Overall, the redundancy is constant (o(1)) after encryption when compared with the unprotected case, which is acceptable in the distributed file system.

### 6.4. Search and Encryption Efficiency Test

**Figure 17:**
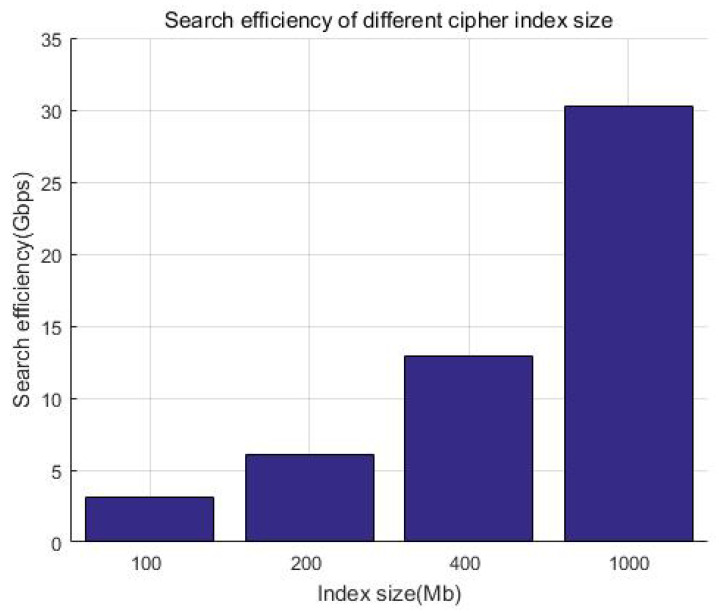
Search efficiency of the system.

**Figure 18:**
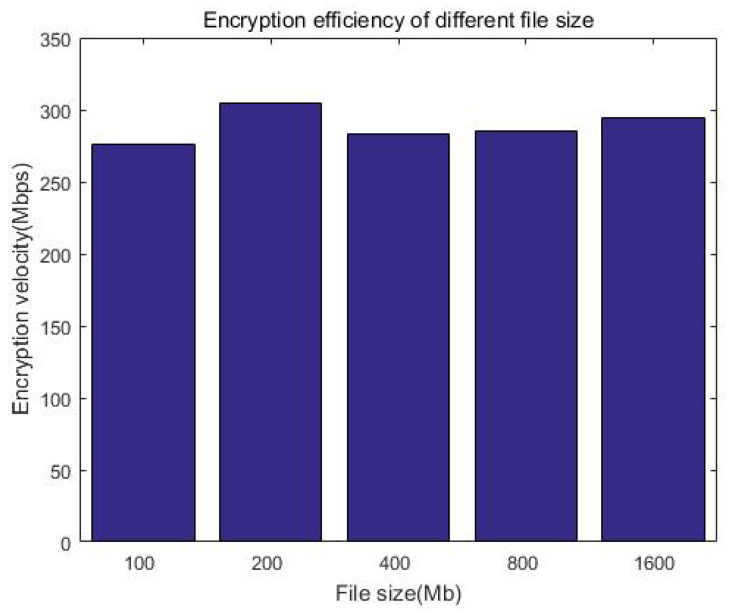
Encryption efficiency of the system.

We consider that the index size of keywords is generally much smaller than the file itself. Considering that Kb-sized documents have a smaller incremental storage overhead compared with big files, without loss of generality, we prepared a 100 MB index for demonstration and tested the time of the search. Figure 17 and Table 6 show that the search efficiency is more than 4000 Mbps, while Figure 18 and Table 7 show that the encryption efficiency is relative stable, around 300 Mbps. It can be seen that whether it is searched or encrypted, even when considering network latency, the efficiency is at the millisecond level and will not affect the user experience after adding security measures.

### 6.5. Security Analysis

Mechanisms such as data encryption, message time stamping, sequence numbers, and signatures can effectively prevent typical protocol attacks, such as man-in-the-middle attacks (“MITM attacks”), replay attacks, tampering attacks, and type attacks. We analyze and demonstrate the security of the protocol against these attacks in the following.

#### 6.5.1. Anti-MITM Attacks

Man-in-the-middle attacks (MITM attacks) mean that the attackers use various means to find and tamper with normal network communication data between participants. The participants generally cannot detect such attacks in these process.

There are many types of man-in-the-middle attacks, and there is still room for expansion, such as SMB session hijacking and DNS spoofing. MITM attacks include information theft, tampering and replay attacks, etc. This section mainly considers the prevention of information theft, while the other types can be found in subsequent subsections.

We use asymmetric encryption algorithms to encrypt the sensitive information in protocols between the client, storage services, key management, and other subsystems. For example, in user registration and authentication, the sender uses the receiver’s public key PK to encrypt sensitive information in the protocol; only the receiver who has the private key SK can decrypt it.

Attackers who intercept this communication link can only obtain the ciphertext. Relying on the secret key’s computation complexity, the protocol ensures that the sensitive information is not leaked. Further, we use HTTPS instead of HTTP to improve the system’s security and prevent attackers from stealing the protocol content.

#### 6.5.2. Anti-Tampering Attacks

Tampering attacks mean that an attacker intercepts the protocol and tampers with the messages between the participants, so as to fake their identity or perform illegal operations.

We introduced signatures in the messages between the client, storage service, key management, and other subsystems. When sending a message, a message digest is generated for the content, and the sender’s private key is used to generate a message signature. The receiver uses the sender’s public key to verify the signature after receiving the message. If the signature fails, the message is discarded. If the content has been tampered with, it will not pass the verification step, thus effectively preventing the tampering attack.

#### 6.5.3. Anti-Replay Attacks

Replay attacks resend the eavesdropped data to the receiver. In some cases, the data transmitted are encrypted, ensuring that eavesdroppers cannot obtain the plaintext. However, if they know the accurate meaning of the data, they can fool the receiving end by sending the data again without knowing the content of the data. For example, some senders simply encrypt authentication information and then transmit it. At this time, although attackers cannot eavesdrop on the passwords, they may intercept the encrypted password and then replay it, thus achieving successful authentication. For another example, suppose a message indicates that the user has withdrawn a deposit. Attackers may intercept and resend this message to steal the deposit.

In the protocol design, we added timestamping and random serial numbers to the sending message, and the signature mechanism ensures that the content including the time and serial numbers cannot be falsified. After receiving the message, the receiver first verifies the signature to ensure that the data have not been falsified. Then, they check the timestamp. If it is not within a valid time range, it is discarded. If the time is valid, the user can check whether the message serial number is novel and if a message with a duplicate “sequence number” has been received, if so, this message will also be discarded. The receiver only saves recent sequence numbers, which are larger than the effective time range to ensure the efficiency of the protocol.

#### 6.5.4. Anti-Type Flaw Attacks

Type flaw attacks attack the protocol by using the type of the protocol message domain, which is not clearly defined, or different domains are expressed in the same way in the implementation. This can destroy the authentication or secrecy of the protocols.

We take the Otway–Rees protocol as an example, which is a symmetric key protocol with a trusted third party. Its purpose is to assign a session key to users A and B. The details of the protocol are described as follows:

1. A→B: M, A, B, ${N_{a},M,A,B}_{K_{as}}$
2. B→S: M, A, B, ${N_{a},M,A,B}_{K_{as}}$, ${N_{b},M,A,B}_{K_{bs}}$
3. S→B: M, ${N_{a},K_{ab}}_{K_{as}}$, ${N_{b},K_{ab}}_{K_{bs}}$
4. B→A: M, ${N_{a},K_{ab}}_{K_{as}}$

Among them, *A* and *B* represent the communication subject. S is the trusted third party, $K_{ab}$ is the session key generated by S, and M represents the number of rounds in the protocol. $N_{a}$ and $N_{b}$ are random numbers used to ensure the freshness of the message through an inquiry/response mechanism. $K_{as}$ and $K_{bs}$ are shared keys of A, B, and S.

One of the type flaw attacks is as follows:

1. A→B: M, A, B, ${N_{a},M,A,B}_{K_{as}}$
2. B→IS: M, A, B, ${N_{a},M,A,B}_{K_{as}}$, ${N_{b},M,A,B}_{K_{bs}}$
3. IS→B: M, ${N_{a},M,A,B}_{K_{as}}$, ${N_{b},M,A,B}_{K_{bs}}$
4. B→A: M, ${N_{a},M,A,B}_{K_{as}}$

Among them, *IS* means that the attacker is impersonating server S. Since ${N_{a},M,A,B}_{K_{as}}$ is processed as a binary string, the attacker uses M,A,B to impersonate $K_{ab}$, which makes the receiver think that the binary string M,A,B is the session key $K_{ab}$, resulting in key leakage, and destroys the authentication of the protocol. In addition to directly using messages in one round of protocols, type flaw attacks can be implemented in parallel sessions of protocols.

There are two main ways to prevent type flaw attacks. One is to avoid similar types of encrypted messages in protocol design, such as changing the order of each field in the ciphertext to ensure that different encryption components have different forms. Another is to add additional information to the encrypted message to identify the type of each part of the message, which was proposed by J. Heather et al. [42]. The additional identifiable information is called a tag.

In our scheme, we use the RESTful API to realize communication between the client, key management, and cipher search server, and message data are in the JSON format, with key–value pairs.

**Figure 19:**
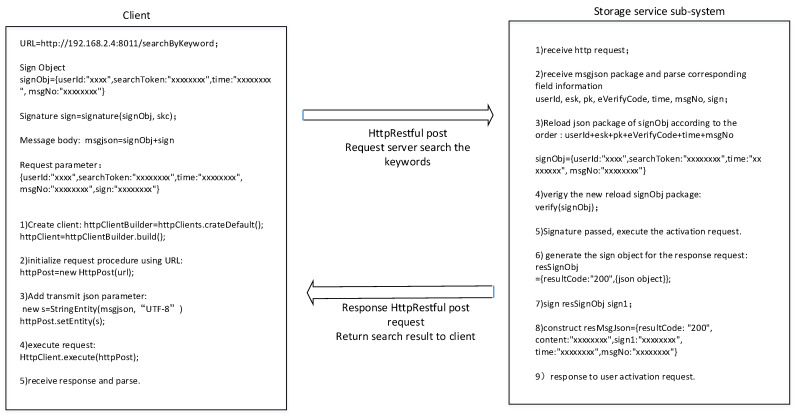
Realization of thecipher search protocol.

The cipher search protocol is an example, as shown in Figure 19. Data field information is explicitly added to the message header and body, which is equivalent to adding message tags. At the same time, we encrypt the sensitive information in the message, and the request parameters of the user’s private key are signed so attackers cannot perform type attacks.

#### 6.5.5. Anti-Internal Adversaries

Our scheme supports users to store the ciphertext and directly query the ciphertext on the distributed file system. The cloud storage provider cannot know the user’s search keywords or the stored plaintext. The scheme allows the data owner to directly share their cloud-stored ciphertext data with others while ensuring data confidentially to service providers. Meanwhile, searchable encryption ensures that the storage provider cannot analyze the file content from user behavior associations. Users can completely master their cloud-stored ciphertext through private keys, and the storage end can only provide services according to the preset rules and not steal the users’ data.

### 6.6. Advantage Analysis

We chose a symmetric-based fine-grained encryption algorithm to deal with massive files compared with the research based on public key encryption. Attribute-based encryption (ABE) is a derivation of public key encryption, such as schemes based on elliptic curve cryptography (ECC) [17,43,44] or the RSA algorithm [45,46]. Their key lengths are different at the same security level according to the National Institute of Standards and Technology (NIST) [47]; for example, a 112-bit symmetrical encryption key has the same strength as a 2048-bit RSA key or a 224-bit ECC key, and thus our strategy is better than others in terms of security and key length.

Table 8 shows a comparison between our EStore and other schemes in terms of fine-grained encryption, fine-grained access control, collaboration, application of big data scenarios, and assumed attacker models. We comprehensively considered the use of a distributed file system, including the confidentiality of storage, accurate authorization, and secure cooperation between users. EStore improved the ability of the existing file system [2,6,7]; further, we took into account both the confidentiality and usage of data and carried out a system evaluation, which has higher practical reference value than other schemes [17,19,21].

## 7. Conclusions

In this paper, we propose a user-friendly encrypted storage scheme, named EStore, based on a distributed file system. Based on the analysis and surveys, we present the highlights as follows:

- We propose an encrypted storage model and architecture for a distributed file system against data leakage, named EStore. We also point out internal and external threats towards the life cycle of data.
- To enable EStore, we designed a fine-grained encryption/decryption algorithm and further designed a searchable encryption algorithm interface to support “direct search on the ciphertext”; in addition, from the perspective of the data life cycle, we implemented six protocols to support the secure usage for a distributed file system.
- Further, we carefully designed key distribution in detail to guard against various adversaries. We also conducted a systematic security analysis to point out the feasibility of our scheme.
- Finally, we evaluated the computation overhead and storage redundancy brought about by encrypted storage. The additional calculation overhead and storage redundancy shows that the price brought about by encryption is acceptable compared with data security. Furthermore, EStore can be easily realized based on a distributed file system in real scenarios.

To the best of our knowledge, EStore is the first encrypted storage scheme based on a distributed file system, and it can efficiently improve the security level, accurately process fine-grained encryption, and enable secure data sharing. The evaluation demonstrates that the scheme can guarantee the storage security and utilization with a minimum price, which is of great significance to the safe use of big data platforms.

## 8. Future Work

Cloud-based data storage has become a paradigm, and its security has far-reaching implications. In the future, we will also investigate other key technologies related to encrypted storage such as secure multi-party, federated learning, and fully homomorphic encryption. We briefly evaluated these technologies shown in Table 9, including their protect phase, strength, and applications. We also noticed that EStore may be improved when combined with other key technologies in the context of data outsourcing. In particular, fully homomorphic encryption (FHE) has a better protective effect on the data process and can adapt to various computing paradigms. Applying FHE in EStore may resist internal attacks brought about by administrators defined as “honest but curious”, which is also an interesting direction for enhancing EStore.

We will continue by exploring more security methods to solve new challenges in future work. First, we will look for an efficient and practical homomorphic encryption method to protect cloud storage. In addition, simplifying the searchable encryption and achieving a better balance between security and efficiency should be considered carefully. We will also attempt to apply our ideas to other scenarios where storage and management need to be protected. For example, P2P networking-based inter-planetary file systems (IPFSs) have become a new trend in the information age. Therefore, exploring the security of the new distributed storage paradigm will have a profound impact on encrypted storage. However, ensuring the balance of security and efficiency will remain a challenge.

## Figures and Tables

**Table 1:** Comparison of the technical route of encrypted storage and secure data sharing.

Reference	Related Work	Encrypted Storage	Secure Data Sharing
Guo et al., 2018 [22]	Search over encrypted data	Edge encryption rather than end devices	None
Zhang et al., 2019 [18]	Broadcast encryption with de-duplication	Emphasizes the de-duplication of the cloud	Based on public key broadcast
Mamta et al., 2021 [16]	Blockchain-assisted fine-grained searchable encryption	Attributed-Based Encryption scheme	Access structure combined with Shamir’s Secret Sharing (based on public keys)
Ning et al., 2022 [20]	Dual access control for cloud data	Symmetric key encryption	None (just anonymity for data owner)
Zhang et al., 2022 [21]	Secure password-protected encryption key for de-duplicated cloud storage	Server-aided message-locked encryption	None
Vanin et al., 2023 [19]	Blockchain-based end-to-end data protection	End-to-end homomorphic encryption	Blockchain-based secure sharing
Gupta et al., 2023 [17]	Multi-authority access control for storage	Attribute-based encryption (based on public key)	Attribute-based access structure (based on public key)

**Table 2:** Notations and descriptions.

Notation	Description
userID	User ID
FK	File key
fileID	File ID
VCode	Verify code
pwd	User password
(sk,pk)	Secret key and corresponding public key pair
msg	Message
$E_{k}\left(msg\right)$	Encrypt msg using k
‖	Concatenation
H	Hash function H: $0,1*\to Z_{p}$
Sign	Sign stands for signing all the previous information
lk	Level key
RK	Root key
r	Random number
sm2/sm3/sm4	Correspond to asymmetric/hash/symmetric type of commercial cryptography

**Table 3:** Resource configuration.

Hardware Requirements	Configuration	Usage
One server for key management	CPU: Intel(R) Xeon(R) Gold 5218 CPU@2.30GHz. Operating system: Ubuntu16.04 LTS	Deployment of key management system
One server for storage service	CPU: Intel(R) Xeon(R) Gold 5218 CPU@2.30GHz. Operating system: Ubuntu16.04 LTS	Deployment of storage service subsystem
Three servers for distributed storage resource	CPU: Intel(R) Xeon(R) Silver 4210 CPU@2.20GHz. Operating system: Ubuntu16.04 LTS	Deployment of HDFS, providing distributed storage resource
Two clients	CPU: Intel(R) Core(TM)i7-6700 CPU@3.40GHz. Operating system: Windows 10	Deployment of the client

**Table 4:** Upload velocity and comparison.

File Size	1 M	5 M	10 M	20 M	40 M	50 M	70 M	100 M
V (upload velocity of calling client SDK)	3.633 MB/s	8.504 MB/s	9.835 MB/s	10.608 MB/s	10.364 MB/s	11.312 MB/s	10.959 MB/s	11.319 MB/s
V0 (direct upload velocity)	3.647 MB/s	8.7 MB/s	10.058 MB/s	10.831 MB/s	10.704 MB/s	11.423 MB/s	11.087 MB/s	11.431 MB/s
V/V0 (average upload speed ratio)	0.996	0.977	0.978	0.979	0.968	0.99	0.988	0.99

**Table 5:** Storage size of the ciphertext and plaintext and their comparison.

File Size	1 M	5 M	10 M	20 M	40 M	50 M	70 M	100 M
S1 (upload size after encryption)	1020.2 MB	1004.14 MB	1002.08 MB	1001.04 MB	1000.52 MB	1000.42 MB	1050.31 MB	1000.21 MB
S0 (direct upload size)	1000 MB	1000 MB	1000 MB	1000 MB	1000 MB	1000 MB	1050 MB	1000 MB
S1/S0-1 (redundancy)	2.0203%	0.4135%	0.2076%	0.104%	0.0521%	0.0417%	0.0298%	0.0208%

**Table 6:** Search efficiency of different index sizes.

**Index Size**	100 Mb	200 Mb	400 Mb	1000 Mb
**Search efficiency**	3.125 Gbps	6.0606 Gbps	12.903 Gbps	30.303 Gbps

**Table 7:** Encryption efficiency of different index sizes.

**File Size**	100 Mb	200 Mb	400 Mb	800 Mb	1600 Mb
**Encryption efficiency**	276.0108 Mbps	304.3091 Mbps	283.6565 Mbps	285.4355 Mbps	294.0944 Mbps

**Table 8:** Comparison with previous work (✓ indicates that the scheme has the corresponding technical capability or adapt to the scene, while the × indicates the opposite).

Author	Fine-Grained Encryption	Fine-Grained Access Control	Active Secure Sharing/Collaboration	Big Data Scenario (Massive Large File Processing)	Assumed Attacker Models
Diaz et al., 2016 [6]	×	×	×	✓	External adversary
Song et al., 2017 [7]	×	×	×	✓	External adversary
Gupta, M et al., 2017 [2]	×	✓	×	✓	External adversary
Zhang et al., 2022 [21]	×	×	×	✓	External adversary and internal adversary
Vanin et al., 2023 [19]	×	×	✓	×	External adversary and internal adversary
Gupta, R et al., 2023 [17]	×	✓	✓	×	External adversary
Ours (EStore)	✓	✓	✓	✓	External adversary and internal adversary

**Table 9:** Key technology evaluation (⋆ represent intensity, and the more ⋆, the stronger the indicator, and the less ⋆, the weaker it is).

Key Technologies	Process Protection	Result Protection	Computation Protection	Precision	Hardware Dependence	Scenarios/Patterns	Actual Used Scene
Secure multi-party computation	⋆⋆⋆⋆⋆	None	⋆⋆	⋆⋆⋆⋆⋆	None	Any computation/decentralization	Auction, salary statistics, key management
Federated learning	⋆⋆⋆	None	⋆⋆⋆	⋆⋆⋆⋆⋆	None	Machine learning modeling/centralization	Key management, federal learning in the financial field
Confidential computation	⋆⋆⋆	None	⋆⋆⋆⋆⋆	⋆⋆⋆⋆⋆	Yes	Any computation/decentralized	Key management, joint modeling, blockchain
Differential privacy	None	Yes	⋆⋆⋆⋆	⋆⋆⋆	None	Any computation/centralization	Google Gboard
Local differential privacy	⋆⋆⋆⋆	Yes	⋆⋆⋆⋆	⋆⋆	None	Data statistics/decentralization and centralization	Google Chrome/iPhone
Fully homomorphic encryption	⋆⋆⋆⋆⋆	None	*⋆*	⋆⋆⋆⋆	None	Any computation/centralization	Unknown

## Data Availability

Not applicable.

## Abbreviations

The following abbreviations are used in this manuscript:

POSIX	Portable Operating System Interface
KMS	Key management system
ACL	Access control list
HDFS	Hadoop distributed file system
PSE	Public key searchable encryption
ORAM	Oblivious random access machine
PIR	Private information retrieval
